# The Concise Guide to PHARMACOLOGY 2015/16: Other ion channels

**DOI:** 10.1111/bph.13351

**Published:** 2015-12-09

**Authors:** Stephen PH Alexander, Eamonn Kelly, Neil Marrion, John A Peters, Helen E Benson, Elena Faccenda, Adam J Pawson, Joanna L Sharman, Christopher Southan, Jamie A Davies, CGTP Collaborators

**Affiliations:** ^1^ School of Biomedical Sciences University of Nottingham Medical School Nottingham, NG7 2UH UK; ^2^ School of Physiology and Pharmacology University of Bristol Bristol BS8 1TD UK; ^3^ School of Physiology and Pharmacology University of Bristol Bristol BS8 1TD UK; ^4^ Neuroscience Division, Medical Education Institute, Ninewells Hospital and Medical School University of Dundee Dundee DD1 9SY UK; ^5^ Centre for Integrative Physiology University of Edinburgh Edinburgh EH8 9XD UK

## Abstract

The Concise Guide to PHARMACOLOGY 2015/16 provides concise overviews of the key properties of over 1750 human drug targets with their pharmacology, plus links to an open access knowledgebase of drug targets and their ligands (www.guidetopharmacology.org), which provides more detailed views of target and ligand properties. The full contents can be found at http://onlinelibrary.wiley.com/doi/10.1111/bph.13351/full. Other ion channels are one of the eight major pharmacological targets into which the Guide is divided, with the others being: G protein‐coupled receptors, ligand‐gated ion channels, voltage‐gated ion channels, nuclear hormone receptors, catalytic receptors, enzymes and transporters. These are presented with nomenclature guidance and summary information on the best available pharmacological tools, alongside key references and suggestions for further reading. The Concise Guide is published in landscape format in order to facilitate comparison of related targets. It is a condensed version of material contemporary to late 2015, which is presented in greater detail and constantly updated on the website www.guidetopharmacology.org, superseding data presented in the previous Guides to Receptors & Channels and the Concise Guide to PHARMACOLOGY 2013/14. It is produced in conjunction with NC‐IUPHAR and provides the official IUPHAR classification and nomenclature for human drug targets, where appropriate. It consolidates information previously curated and displayed separately in IUPHAR‐DB and GRAC and provides a permanent, citable, point‐in‐time record that will survive database updates.

## Conflict of interest

The authors state that there are no conflicts of interest to declare.

## Family structure

5943 Aquaporins

5944 Chloride channels

5944 ClC family

5947 CFTR

5948 Calcium activated chloride channel

5949 Maxi chloride channel

5950 Volume regulated chloride channels

5952 Connexins and Pannexins

5954 Sodium leak channel, non‐selective

## Aquaporins

### Overview

Aquaporins and aquaglyceroporins are membrane channels that allow the permeation of water and certain other small solutes across the cell membrane. Since the isolation and cloning of the first aquaporin (AQP1) [77], 12 additional members of the family have been identified, although little is known about the functional properties of two of these (*AQP11*; Q8NBQ7 and *AQP12A*; Q8IXF9). The other 11 aquaporins can be divided into two families (aquaporins and aquaglyceroporins) depending on whether they are permeable to glycerol[41]. One or more members of this family of proteins have been found to be expressed in almost all tissues of the body. Individual AQP subunits have six transmembrane domains with an inverted symmetry between the first three and last three domains [15]. Functional AQPs exist as tetramers but, unusually, each subunit contains a separate pore, so each channel has four pores.

**Table:** 

Nomenclature	AQP0	AQP1	AQP2	AQP3	AQP4	AQP5
HGNC, UniProt	*MIP*, P30301	*AQP1*, P29972	*AQP2*, P41181	*AQP3*, Q92482	*AQP4*, P55087	*AQP5*, P55064
Permeability	water (low)	water (high)	water (high)	water (high), glycerol	water (high)	water (high)
Endogenous activators	–	cyclic GMP	–	–	–	–
Inhibitors	Hg^2+^	Ag^+^, Hg^2+^, tetraethylammonium	Hg^2+^	Hg^2+^ (also inhibited by acid pH)	–	Hg^2+^
Comments	–	–	–	AQP3 is also inhibited by acid pH	AQP4 is inhibited by PKC activation	–

**Table:** 

Nomenclature	AQP6	AQP7	AQP8	AQP9	AQP10
HGNC, UniProt	*AQP6*, Q13520	*AQP7*, O14520	*AQP8*, O94778	*AQP9*, O43315	*AQP10*, Q96PS8
Permeability	water (low), anions	water (high), glycerol	water (high)	water (low), glycerol	water (low), glycerol
Inhibitors	Hg^2+^	Hg^2+^	Hg^2+^	Hg^2+^, phloretin	Hg^2+^
Comments	AQP6 is an intracellular channel permeable to anions as well as water [106]	–	–	–	–

### Further Reading

Amiry‐Moghaddam M *et al*. (2003) The molecular basis of water transport in the brain. *Nat. Rev. Neurosci.* **4**: 991‐1001 [PMID:14682361]

Carbrey JM *et al*. (2009) Discovery of the aquaporins and development of the field. *Handb Exp Pharmacol* 3‐28 [PMID:19096770]

Castle NA. (2005) Aquaporins as targets for drug discovery. *Drug Discov. Today* **10**: 485‐93 [PMID:15809194]

Kimelberg HK. (2004) Water homeostasis in the brain: basic concepts. *Neuroscience* **129**: 851‐60 [PMID:15561403]

King LS *et al*. (2004) From structure to disease: the evolving tale of aquaporin biology. *Nat. Rev. Mol. Cell Biol.* **5**: 687‐98 [PMID:15340377]

Rojek A *et al*. (2008) A current view of the mammalian aquaglyceroporins. *Annu. Rev. Physiol.* **70**: 301‐27 [PMID:17961083]

Verkman AS. (2009) Aquaporins: translating bench research to human disease. *J. Exp. Biol.* **212**: 1707‐15 [PMID:19448080]

## Chloride channels

### Overview

Chloride channels are a functionally and structurally diverse group of anion selective channels involved in various processes including the regulation of the excitability of neurones, skeletal, cardiac and smooth muscle, cell volume regulation, transepithelial salt transport, the acidification of internal and extracellular compartments, the cell cycle and apoptosis (reviewed in [22]). Excluding the transmitter‐gated GABA_A_ and glycine receptors (see separate tables), well characterised chloride channels can be classified as certain members of the voltage‐sensitive ClC subfamily, calcium‐activated channels, high (maxi) conductance channels, the cystic fibrosis transmembrane conductance regulator (CFTR) and volume regulated channels [101]. No official recommendation exists regarding the classification of chloride channels. Functional chloride channels that have been cloned from, or characterised within, mammalian tissues are listed with the exception of several classes of intracellular channels (*e.g.* CLIC) that are reviewed in [26].

## ClC family

### Overview

The mammalian ClC family (reviewed in [2, 16, 22, 24, 40]) contains 9 members that fall, on the basis of sequence homology, into three groups; ClC‐1, ClC‐2, hClC‐Ka (rClC‐K1) and hClC‐Kb (rClC‐K2); ClC‐3 to ClC‐5, and ClC‐6 and ‐7. ClC‐1 and ClC‐2 are plasma membrane chloride channels. ClC‐Ka and ClC‐Kb are also plasma membrane channels (largely expressed in the kidney and inner ear) when associated with barttin (*BSND*, Q8WZ55), a 320 amino acid 2TM protein [27]. The localisation of the remaining members of the ClC family is likely to be predominantly intracellular *in vivo*, although they may traffic to the plasma membrane in overexpression systems. Numerous recent reports indicate that ClC‐4, ClC‐5, ClC‐6 and ClC‐7 (and by inference ClC‐3) function as Cl^‐^/H^+^ antiporters (secondary active transport), rather than classical Cl^‐^ channels [34, 48, 62, 73, 87]; reviewed in [2, 79]). It has recently been reported that the activity of ClC‐5 as a Cl^‐^/H^+^ exchanger is important for renal endocytosis [64]. Alternative splicing increases the structural diversity within the ClC family. The crystal structure of two bacterial ClC proteins has been described [25] and a eukaryotic ClC transporter (CmCLC) has recently been described at 3.5 Å resolution [30]. Each ClC subunit, with a complex topology of 18 intramembrane segments, contributes a single pore to a dimeric ‘double‐barrelled’ ClC channel that contains two independently gated pores, confirming the predictions of previous functional and structural investigations (reviewed in [16, 24, 40, 79]). As found for ClC‐4, ClC‐5, ClC‐6 and ClC‐7, the prokaryotic ClC homologue (ClC‐ec1) and CmCLC function as H^+^/Cl antiporters, rather than as ion channels [1, 30]. The generation of monomers from dimeric ClC‐ec1 has firmly established that each ClC subunit is a functional unit for transport and that cross‐subunit interaction is not required for Cl^‐^/H^+^ exchange in ClC transporters [81].

**Table:** 

Nomenclature	ClC‐1	ClC‐2	ClC‐Ka	ClC‐Kb
HGNC, UniProt	*CLCN1*, P35523	*CLCN2*, P51788	*CLCNKA*, P51800	*CLCNKB*, P51801
Functional Characteristics	*γ* = 1‐1.5 pS; voltage‐activated (depolarization) (by fast gating of single protopores and a slower common gate allowing both pores to open simultaneously); inwardly rectifying; incomplete deactivation upon repolarization, ATP binding to cytoplasmic cystathionine *β*‐synthetase related (CBS) domains inhibits ClC‐1 (by closure of the common gate), depending on its redox status	*γ* = 2‐3 pS; voltage‐activated by membrane hyperpolarization by fast protopore and slow cooperative gating; channels only open negative to E_Cl_ resulting in steady‐state inward rectification; voltage dependence modulated by permeant anions; activated by cell swelling, PKA, and weak extracellular acidosis; potentiated by SGK1; inhibited by phosphorylation by p34(cdc2)/cyclin B; cell surface expression and activity increased by association with Hsp90	*γ* = 26 pS; linear current‐voltage relationship except at very negative potentials; no time dependence; inhibited by extracellular protons (p*K* = 7.1); potentiated by extracellular Ca^2+^	Bidirectional rectification; no time dependence; inhibited by extracellular protons; potentiated by extracellular Ca^2+^
Endogenous activators	–	arachidonic acid	–	–
Activators	–	lubiprostone, omeprazole	niflumic acid (pEC_50_ 3–5)	niflumic acid (pEC_50_ 3–5)
Channel blockers	9‐anthroic acid, *S*‐(‐)CPB, *S*‐(‐)CPP, Cd^2+^, Zn^2+^, fenofibric acid, niflumic acid	GaTx2 (p*K*_d_ 10.8) [*voltage dependent* ‐100mV], Cd^2+^, NPPB, Zn^2+^, diphenylamine‐2‐carboxylic acid	3‐phenyl‐CPP, DIDS, niflumic acid	3‐phenyl‐CPP, DIDS
Comments	CIC‐1 is constitutively active	CIC‐2 is also activated by amidation	CIC‐Ka is constitutively active (when co‐expressed with barttin), and can be blocked by benzofuran derivatives	CIC‐Kb is constitutively active (when co‐expressed with barttin), and can be blocked by benzofuran derivatives

**Table:** 

Nomenclature	ClC‐3	ClC‐4	ClC‐5	ClC‐6	ClC‐7
HGNC, UniProt	*CLCN3*, P51790	*CLCN4*, P51793	*CLCN5*, P51795	*CLCN6*, P51797	*CLCN7*, P51798
Functional Characteristics	Cl^‐^/H^+^ antiporter [58]; pronounced outward rectification; slow activation, fast deactivation; activity enhanced by CaM kinase II; inhibited by intracellular Ins(3,4,5,6)P4 and extracellular acidosis	Cl^‐^/H^+^ antiporter (2Cl^‐^:1H^+^) [3, 73, 87]; extreme outward rectification; voltage‐dependent gating with midpoint of activation at +73 mV [67]; rapid activation and deactivation; inhibited by extracellular acidosis; non‐hydrolytic nucleotide binding required for full activity	Cl^‐^/H^+^ antiporter (2Cl^‐^:1H^+^) [73, 87, 94, 109]; extreme outward rectification; voltage‐dependent gating with midpoint of activation of 116.0 mV; rapid activation and deactivation; potentiated and inhibited by intracellular and extracellular acidosis, respectively; ATP binding to cytoplasmic cystathionine *β*‐synthetase related (CBS) domains activates ClC‐5	Cl^‐^/H^+^ antiporter (2Cl^‐^:1H^+^) [62]; outward rectification, rapid activation and deactivation	Cl^‐^/H^+^ antiporter (2Cl^‐^:1H^+^) [34, 48, 90]; strong outward rectification; voltage‐dependent gating with a threshold more positive than + 20 mV; very slow activation and deactivation
Channel blockers	phloretin (pIC_50_ 4.5)	Zn^2+^ (pIC_50_ 4.3) [68], Cd^2+^ (pIC_50_ 4.2) [68]	–	DIDS (pIC_50_ 3)	DIDS (pIC_50_ 4.4) [90], NS5818 (pIC_50_ 4.3) [90], NPPB (pIC_50_ 3.8) [90]
Comments	insensitive to the channel blockers DIDS, NPPB and tamoxifen (10 *μ*M)	–	insensitive to the channel blockers DIDS (1 mM), diphenylamine‐2‐carboxylic acid (1 mM), 9‐anthroic acid (2 mM), NPPB (0.5 mM) and niflumic acid (1 mM)	–	active when co‐expressed with Ostm1

### Comments

ClC channels display the permeability sequence Cl^‐^> Br^‐^> I^‐^ (at physiological pH). ClC‐1 has significant opening probability at resting membrane potential, accounting for 75% of the membrane conductance at rest in skeletal muscle, and is important for stabilization of the membrane potential. *S*‐(‐)CPP, 9‐anthroic acid and niflumic acid act intracellularly and exhibit a strongly voltage‐dependent block with strong inhibition at negative voltages and relief of block at depolarized potentials ([49] and reviewed in [78]). Inhibition of ClC‐2 by the peptide GaTx2, from *Leiurus quinquestriatus herbareus* venom, is likely to occur through inhibition of channel gating, rather than direct open channel blockade [98]. Although ClC‐2 can be activated by cell swelling, it does not correspond to the VRAC channel (see below). Alternative potential physiological functions for ClC‐2 are reviewed in [76]. Functional expression of human ClC‐Ka and ClC‐Kb requires the presence of barttin [27, 88] reviewed in [29]. The properties of ClC‐Ka/barttin and ClC‐Kb/barttin tabulated are those observed in mammalian expression systems: in oocytes the channels display time‐ and voltage‐dependent gating. The rodent homologue (ClC‐K1) of ClC‐Ka demonstrates limited expression as a homomer, but its function is enhanced by barttin which increases both channel opening probablility in the physiological range of potentials [27, 32, 88] reviewed in [29]). ClC‐Ka is approximately 5 to 6‐fold more sensitive to block by 3‐phenyl‐CPP and DIDS than ClC‐Kb, while newly synthesized benzofuran derivatives showed the same blocking affinity (<10 *μ*M) on both CLC‐K isoforms [50]. The biophysical and pharmacological properties of ClC‐3, and the relationship of the protein to the endogenous volume‐regulated anion channel(s) VRAC [4, 36] are controversial and further complicated by the possibility that ClC‐3 may function as both a Cl^‐^/H^+^ exchanger and an ion channel [4, 73, 104]. The functional properties tabulated are those most consistent with the close structural relationship between ClC‐3, ClC‐4 and ClC‐5. Activation of heterologously expressed ClC‐3 by cell swelling in response to hypotonic solutions is disputed, as are many other aspects of its regulation. Dependent upon the predominant extracellular anion (*e.g.* SCN^‐^ versus Cl^‐^), CIC‐4 can operate in two transport modes: a slippage mode in which behaves as an ion channel and an exchanger mode in which unitary transport rate is 10‐fold lower [3]. Similar findings have been made for ClC‐5 [108]. ClC‐7 associates with a *β* subunit, Ostm1, which increases the stability of the former [45] and is essential for its function [48].

## CFTR

### Overview

CFTR, a 12TM, ABC transporter‐type protein, is a cAMP‐regulated epithelial cell membrane Cl^‐^ channel involved in normal fluid transport across various epithelia. Of the 1700 mutations identified in CFTR, the most common is the deletion mutant *Δ*F508 (a class 2 mutation) which results in impaired trafficking of CFTR and reduces its incorporation into the plasma membrane causing cystic fibrosis (reviewed in [18]). Channels carrying the *Δ*F508 mutation that do traffic to the plasma membrane demonstrate gating defects. Thus, pharmacological restoration of the function of the *Δ*F508 mutant would require a compound that embodies ‘corrector’ (*i.e.* facilitates folding and trafficking to the cell surface) and ‘potentiator’ (*i.e.* promotes opening of channels at the cell surface) activities [18]. In addition to acting as an anion channel *per se*, CFTR may act as a regulator of several other conductances including inhibition of the epithelial Na channel (ENaC), calcium activated chloride channels (CaCC) and volume regulated anion channel (VRAC), activation of the outwardly rectifying chloride channel (ORCC), and enhancement of the sulphonylurea sensitivity of the renal outer medullary potassium channel (ROMK2), (reviewed in [63]). CFTR also regulates TRPV4, which provides the Ca^2+^ signal for regulatory volume decrease in airway epithelia [6]. The activities of CFTR and the chloride‐bicarbonate exchangers SLC26A3 (DRA) and SLC26A6 (PAT1) are mutually enhanced by a physical association between the regulatory (R) domain of CFTR and the STAS domain of the SCL26 transporters, an effect facilitated by PKA‐mediated phosphorylation of the R domain of CFTR [42].

**Table:** 

Nomenclature	CFTR
HGNC, UniProt	*CFTR*, P13569
Functional Characteristics	*γ* = 6‐10 pS; permeability sequence = Br^‐^≥ Cl^‐^> I^‐^> F^‐^, (P_I_/P_Cl_ = 0.1‐0.85); slight outward rectification; phosphorylation necessary for activation by ATP binding at binding nucleotide binding domains (NBD)1 and 2; positively regulated by PKC and PKGII (tissue specific); regulated by several interacting proteins including syntaxin 1A, Munc18 and PDZ domain proteins such as NHERF (EBP50) and CAP70
Activators	felodipine (Potentiation) (p*K*_i_ 8.4) [71], CBIQ (Potentiation), NS004 (Potentiation), UCCF‐029 (Potentiation), UCCF‐339 (Potentiation), UCCF‐853 (Potentiation), apigenin (Potentiation), capsaicin (Potentiation), genistein (Potentiation), ivacaftor (Potentiation), nimodipine (Potentiation), phenylglycine‐01 (Potentiation), sulfonamide‐01 (Potentiation)
Selective inhibitors	crofelemer (pIC_50_ 5.2) [99]
Channel blockers	glibenclamide (p*K*_i_ 4.7) [91], intracellular CFTR_inh_‐172 (intracellular application prolongs mean closed time), GaTx1, extracellular GlyH‐101
Comments	UCCF‐339, UCCF‐029, apigenin and genistein are examples of flavones. UCCF‐853 and NS004 are examples of benzimidazolones. CBIQ is an example of a benzoquinoline. felodipine and nimodipine are examples of 1,4‐dihydropyridines. phenylglycine‐01 is an example of a phenylglycine. sulfonamide‐01 is an example of a sulfonamide. Malonic acid hydrazide conjugates are also CFTR channel blockers (see Verkman and Galietta, 2009 [101])

### Comments

In addition to the agents listed in the table, the novel small molecule, ataluren, induces translational read through of nonsense mutations in CFTR (reviewed in [93]). Corrector compounds that aid the folding of DF508CFTR to increase the amount of protein expressed and potentially delivered to the cell surface include VX‐532 (which is also a potentiator), VRT‐325, KM11060, Corr‐3a and Corr‐4a see [101] for details and structures of Corr‐3a and Corr‐4a). Inhibition of CFTR by intracellular application of the peptide GaTx1, from *Leiurus quinquestriatus herbareus* venom, occurs preferentially for the closed state of the channel [33]. CFTR contains two cytoplasmic nucleotide binding domains (NBDs) that bind ATP. A single open‐closing cycle is hypothesised to involve, in sequence: binding of ATP at the N‐terminal NBD1, ATP binding to the C‐terminal NBD2 leading to the formation of an intramolecular NBD1‐NBD2 dimer associated with the open state, and subsequent ATP hydrolysis at NBD2 facilitating dissociation of the dimer and channel closing, and the initiation of a new gating cycle [5, 59]. Phosphorylation by PKA at sites within a cytoplasmic regulatory (R) domain facilitates the interaction of the two NBD domains. PKC (and PKGII within intestinal epithelial cells via guanylinstimulated cyclic GMP formation) positively regulate CFTR activity.

## Calcium activated chloride channel

### Overview

Chloride channels activated by intracellular calcium (CaCC) are widely expressed in excitable and non‐excitable cells where they perform diverse functions [37]. The molecular nature of CaCC has been uncertain with both *CLCA*, *TWEETY* and *BEST* genes having been considered as likely candidates [22, 38, 51]. It is now accepted that CLCA expression products are unlikely to form channels *per se* and probably function as cell adhesion proteins, or are secreted [70]. Similarly, *TWEETY* gene products do not recapictulate the properties of endogenous CaCC. The bestrophins encoded by genes *BEST1‐4* have a topology more consistent with ion channels [38] and form chloride channels that are activated by physiological concentrations of Ca^2+^, but whether such activation is direct is not known [38]. However, currents generated by bestrophin over‐expression do not resemble native CaCC currents. The evidence for and against bestrophin proteins forming CaCC is critically reviewed by Duran *et al.* [22]. Recently, a new gene family, TMEM16 (anoctamin) consisting of 10 members (TMEM16A‐K; anoctamin 1‐10) has been identified and there is firm evidence that some of these members form chloride channels [21, 43]. TMEM16A (anoctamin 1; Ano 1) produces Ca^2+^‐activated Cl^‐^ currents with kinetics similar to native CaCC currents recorded from different cell types [14, 82, 89, 105]. Knockdown of TMEM16A greatly reduces currents mediated by calcium‐activated chloride channels in submandibular gland cells [105] and smooth muscle cells from pulmonary artery [55]. In TMEM16A^(‐/‐)^ mice secretion of Ca^2+^‐dependent Cl^‐^secretion by several epithelia is reduced [69, 82]. Alternative splicing regulates the voltage‐ and Ca^2+^‐ dependence of TMEM16A and such processing may be tissue‐specific manner and thus contribute to functional diversity [31]. There are also reports that TMEM16B (anoctamin 2; Ano 2) supports CaCC activity (*e.g*.[74]) and in TMEM16B^(‐/‐)^ mice Ca‐activated Cl‐ currents in the main olfactory epithelium (MOE) and in the vomeronasal organ are virtually absent[11] .

**Table:** 

Nomenclature	CaCC
HGNC, UniProt	*ANO1*, Q5XXA6
Functional Characteristics	*γ* = 0.5‐5 pS; permeability sequence, SCN^‐^> NO_3_^‐^> I^‐^> Br^‐^> Cl^‐^> F^‐^; relative permeability of SCN^‐^:Cl^‐^∼8. I^‐^:Cl^‐^∼3, aspartate:Cl^‐^∼0.15, outward rectification (decreased by increasing [Ca^2+^]_i_); sensitivity to activation by [Ca^2+^]_i_ decreased at hyperpolarized potentials; slow activation at positive potentials (accelerated by increasing [Ca^2+^]_i_); rapid deactivation at negative potentials, deactivation kinetics modulated by anions binding to an external site; modulated by redox status
Endogenous activators	intracellular Ca^2+^
Selective inhibitors	crofelemer (pIC_50_ 5.2) [99]
Endogenous channel blockers	Ins(3,4,5,6)P_4_
Channel blockers	9‐anthroic acid, DCDPC, DIDS, NPPB, SITS, flufenamic acid, fluoxetine, mibefradil, niflumic acid, tannic acid

### Comments

Blockade of I_Cl(Ca)_ by niflumic acid, DIDS and 9‐anthroic acid is voltage‐dependent whereas block by NPPB is voltage‐independent [37]. Extracellular niflumic acid; DCDPC and 9‐anthroic acid (but not DIDS) exert a complex effect upon I_Cl(Ca)_ in vascular smooth muscle, enhancing and inhibiting inwardly and outwardly directed currents in a manner dependent upon [Ca^2+^]_i_(see [46] for summary). Considerable crossover in pharmacology with large conductance Ca^2+^‐activated K^+^ channels also exists (see [35] for overview). Two novel compounds, CaCC_inh_‐A01 and CaCC_inh_‐B01 have recently been identified as blockers of calcium‐activated chloride channels in T84 human intestinal epithelial cells [19] for structures). Significantly, other novel compounds totally block currents mediated by TMEM116A, but have only a modest effect upon total current mediated by CaCC native to T84 cells or human bronchial epithelial cells, suggesting that TMEM16A is not the predominant CaCC in such cells [61]. CaMKII modulates CaCC in a tissue dependent manner (reviewed by [37, 46]). CaMKII inhibitors block activation of I_Cl(Ca)_ in T84 cells but have no effect in parotid acinar cells. In tracheal and arterial smooth muscle cells, but not portal vein myocytes, inhibition of CaMKII reduces inactivation of I_Cl(Ca)_. Intracellular Ins(3,4,5,6)P_4_ may act as an endogenous negative regulator of CaCC channels activated by Ca^2+^, or CaMKII. Smooth muscle CaCC are also regulated positively by Ca^2+^‐dependent phosphatase, calcineurin (see [46] for summary).

## Maxi chloride channel

### Overview

Maxi Cl^‐^ channels are high conductance, anion selective, channels initially characterised in skeletal muscle and subsequently found in many cell types including neurones, glia, cardiac muscle, lymphocytes, secreting and absorbing epithelia, macula densa cells of the kidney and human placenta syncytiotrophoblasts [84]. The physiological significance of the maxi Cl^‐^ channel is uncertain, but roles in cell volume regulation and apoptosis have been claimed. Evidence suggests a role for maxi Cl^‐^ channels as a conductive pathway in the swelling‐induced release of ATP from mouse mammary C127i cells that may be important for autocrine and paracrine signalling by purines [23, 83]. A similar channel mediates ATP release from macula densa cells within the thick ascending of the loop of Henle in response to changes in luminal NaCl concentration [9]. A family of human high conductance Cl^‐^ channels (TTYH1‐3) that resemble Maxi Cl^‐^ channels has been cloned [95], but alternatively, Maxi Cl^‐^ channels have also been suggested to correspond to the voltage‐dependent anion channel, VDAC, expressed at the plasma membrane [7, 65].

**Table:** 

Nomenclature	Maxi Cl^‐^
Functional Characteristics	*γ* = 280‐430 pS (main state); permeability sequence, I > Br > Cl > F > gluconate (P_CI_P_Cl_ = ∼1.5); ATP is a voltage dependent permeant blocker of single channel activity (P_ATP_/P_Cl_ = 0.08‐0.1); channel activity increased by patch‐excision; channel opening probability (at steady‐state) maximal within approximately ± 20 mV of 0 mV, opening probability decreased at more negative and (commonly) positive potentials yielding a bell‐shaped curve; channel conductance and opening probability regulated by annexin 6
Activators	cytosolic GTP*γ*S, extracellular chlorpromazine, extracellular tamoxifen, extracellular toremifene, extracellular triflupromazine
Endogenous channel blockers	intracellular arachidonic acid
Channel blockers	DIDS (pIC_50_ 4.4) [90], extracellular Zn^2+^ (pIC_50_ 4.3) [68], NPPB (pIC_50_ 3.8) [90], extracellular Gd^3+^, SITS, diphenylamine‐2‐carboxylic acid
Comments	Maxi Cl^‐^ is also activated by G protein‐coupled receptors and cell swelling. tamoxifen and toremifene are examples of triphenylethylene anti‐oestrogens

### Comments

Differing ionic conditions may contribute to variable estimates of *γ* reported in the literature. Inhibition by arachidonic acid (and cis‐unsaturated fatty acids) is voltage‐independent, occurs at an intracellular site, and involves both channel shut down (K*_d_* = 4‐5 *μ*M) and a reduction of *γ*(K*_d_* = 13‐14 *μ*M). Blockade of channel activity by SITS, DIDS, Gd^3+^ and arachidonic acid is paralleled by decreased swelling‐induced release of ATP[23, 83]. Channel activation by anti‐oestrogens in whole cell recordings requires the presence of intracellular nucleotides and is prevented by pre‐treatment with 17*β*‐estradiol, bucladesine, or intracellular dialysis with GDP*β*S [20]. Activation by tamoxifen is suppressed by low concentrations of okadaic acid, suggesting that a dephosphorylation event by protein phosphatase PP2A occurs in the activation pathway [20]. In contrast, 17*β*‐estradiol and tamoxifen appear to directly inhibit the maxi Cl^‐^ channel of human placenta reconstituted into giant liposomes and recorded in excised patches [80].

## Volume regulated chloride channels

### Overview

Volume activated chloride channels (also termed VSOAC, volume‐sensitive organic osmolyte/anion channel; VRC, volume regulated channel and VSOR, volume expansion‐sensing outwardly rectifying anion channel) participate in regulatory volume decrease (RVD) in response to cell swelling. VRAC may also be important for several other processes including the regulation of membrane excitability, transcellular Cl^‐^ transport, angiogenesis, cell proliferation, necrosis, apoptosis, glutamate release from astrocytes, insulin(*INS*, P01308) release from pancreatic *β* cells and resistance to the anti‐cancer drug, cisplatin(reviewed by [10, 60, 63, 66]). VRAC may not be a single entity, but may instead represent a number of different channels that are expressed to a variable extent in different tissues and are differentially activated by cell swelling. In addition to ClC‐3 expression products (see above) several former VRAC candidates including *MDR1* (ABCB1, P‐glycoprotein), Icln, Band 3 anion exchanger (SLC4A1) and phospholemman are also no longer considered likely to fulfil this function (see reviews [63, 86]).

**Table:** 

Nomenclature	VRAC
Functional Characteristics	*γ* = 10‐20 pS (negative potentials), 50‐90 pS (positive potentials); permeability sequence SCN > I > NO_3_‐ >Br^‐^> Cl^‐^> F^‐^> gluconate; outward rectification due to voltage dependence of *γ*; inactivates at positive potentials in many, but not all, cell types; time dependent inactivation at positive potentials; intracellular ionic strength modulates sensitivity to cell swelling and rate of channel activation; rate of swelling‐induced activation is modulated by intracellular ATP concentration; ATP dependence is independent of hydrolysis and modulated by rate of cell swelling; inhibited by increased intracellular free Mg^2+^ concentration; swelling induced activation of several intracellular signalling cascades may be permissive of, but not essential to, the activation of VRAC including: the Rho‐Rho kinase‐MLCK; Ras‐Raf‐MEK‐ERK; PIK3‐NOX‐H_2_O_2_ and Src‐PLC*γ*‐Ca^2+^ pathways; regulation by PKC*α* required for optimal activity; cholesterol depletion enhances activity; activated by direct stretch of *β*1‐integrin
Activators	GTP*γ*S
Endogenous channel blockers	intracellular Mg^2+^, arachidonic acid
Channel blockers	1,9‐dideoxyforskolin, 9‐anthroic acid, DCPIB, DIDS, IAA‐94, NPPB, NS3728, carbenoxolone, clomiphene, diBA‐(5)‐C4, gossypol, mefloquine, mibefradil, nafoxidine, nordihydroguiaretic acid, quinidine, quinine, tamoxifen
Comments	VRAC is also activated by cell swelling and low intracellular ionic strength. VRAC is also blocked by chromones, extracellular nucleotides and nucleoside analogues

### Comments

In addition to conducting monovalent anions, in many cell types the activation of VRAC by a hypotonic stimulus can allow the efflux of organic osmolytes such as amino acids and polyols that may contribute to RVD.

### Comments: Other chloride channels

In addition to some intracellular chloride channels that are not considered here, plasma membrane channels other than those listed have been functionally described. Many cells and tissues contain outwardly rectifying chloride channels (ORCC) that may correspond to VRAC active under isotonic conditions. A cyclic AMP‐activated Cl^‐^ channel that does not correspond to CFTR has been described in intestinal Paneth cells [100]. A Cl channel activated by cyclic GMP with a dependence on raised intracellular Ca^2+^ has been recorded in various vascular smooth muscle cells types, which has a pharmacology and biophysical characteristics very different from the ‘conventional’ CaCC [56, 75]. It has been proposed that bestrophin‐3(*BEST3*, Q8N1M1) is an essential component of the cyclic GMP‐activated channel [57]. A proton‐activated, outwardly rectifying anion channel has also been described [44].

### Further Reading

Accardi A *et al*. (2010) CLC channels and transporters: proteins with borderline personalities. *Biochim. Biophys. Acta* **1798**: 1457‐64 [PMID:20188062]

Alekov AK *et al*. (2008) Anion channels: regulation of ClC‐3 by an orphan second messenger. *Curr. Biol.* **18**: R1061‐4 [PMID:19036336]

Aleksandrov AA *et al*. (2007) CFTR (ABCC7) is a hydrolyzable‐ligand‐gated channel. *Pflugers Arch.* **453**: 693‐702 [PMID:17021796]

Amaral MD *et al*. (2007) Molecular targeting of CFTR as a therapeutic approach to cystic fibrosis. *Trends Pharmacol. Sci.* **28**: 334‐41 [PMID:17573123]

Aromataris EC *et al*. (2006) ClC‐1 chloride channel: Matching its properties to a role in skeletal muscle. *Clin. Exp. Pharmacol. Physiol.* **33**: 1118‐23 [PMID:17042925]

Ashlock MA *et al*. (2011) Therapeutics development for cystic fibrosis: a successful model for a multisystem genetic disease. *Annu. Rev. Med.* **62**: 107‐25 [PMID:21226613]

Best L *et al*. (2010) Electrical activity in pancreatic islet cells: The VRAC hypothesis. *Islets* **2**: 59‐64 [PMID:21099297]

Chen TY. (2005) Structure and function of clc channels. *Annu. Rev. Physiol.* **67**: 809‐39 [PMID:15709979]

Chen TY *et al*. (2008) CLC‐0 and CFTR: chloride channels evolved from transporters. *Physiol. Rev.* **88**: 351‐87 [PMID:18391167]

Cuthbert AW. (2011) New horizons in the treatment of cystic fibrosis. *Br. J. Pharmacol.* **163**: 173‐83 [PMID:21108631]

Duan D. (2009) Phenomics of cardiac chloride channels: the systematic study of chloride channel function in the heart. *J. Physiol. (Lond.)* **587**: 2163‐77 [PMID:19171656]

Duan DD. (2011) The ClC‐3 chloride channels in cardiovascular disease. *Acta Pharmacol. Sin.* **32**: 675‐84 [PMID:21602838]

Duran C *et al*. (2011) Physiological roles and diseases of Tmem16/Anoctamin proteins: are they all chloride channels? *Acta Pharmacol. Sin.* **32**: 685‐92 [PMID:21642943]

Duran C *et al*. (2010) Chloride channels: often enigmatic, rarely predictable. *Annu. Rev. Physiol.* **72**: 95‐121 [PMID:19827947]

Dutzler R. (2007) A structural perspective on ClC channel and transporter function. *FEBS Lett.* **581**: 2839‐44 [PMID:17452037]

Edwards JC *et al*. (2010) Chloride channels of intracellular membranes. *FEBS Lett.* **584**: 2102‐11 [PMID:20100480]

Fahlke C *et al*. (2010) Physiology and pathophysiology of ClC‐K/barttin channels. *Front Physiol* **1**: 155 [PMID:21423394]

Ferrera L *et al*. (2010) TMEM16A protein: a new identity for Ca(2+)‐dependent Cl^−^ channels. *Physiology (Bethesda)* **25**: 357‐63 [PMID:21186280]

Gadsby DC *et al*. (2006) The ABC protein turned chloride channel whose failure causes cystic fibrosis. *Nature* **440**: 477‐83 [PMID:16554808]

Galietta LJ. (2009) The TMEM16 protein family: a new class of chloride channels? *Biophys. J.* **97**: 3047‐53 [PMID:20006941]

Greenwood IA *et al*. (2007) Overlapping pharmacology of Ca2+‐activated Cl‐ and K+ channels. *Trends Pharmacol. Sci.* **28**: 1‐5 [PMID:17150263]

Guan YY *et al*. (2006) The ClC‐3 Cl‐ channel in cell volume regulation, proliferation and apoptosis in vascular smooth muscle cells. *Trends Pharmacol. Sci.* **27**: 290‐6 [PMID:16697056]

Hartzell C *et al*. (2005) Calcium‐activated chloride channels. *Annu. Rev. Physiol.* **67**: 719‐58 [PMID:15709976]

Hartzell HC *et al*. (2008) Molecular physiology of bestrophins: multifunctional membrane proteins linked to best disease and other retinopathies. *Physiol. Rev.* **88**: 639‐72 [PMID:18391176]

Jentsch TJ. (2008) CLC chloride channels and transporters: from genes to protein structure, pathology and physiology. *Crit. Rev. Biochem. Mol. Biol.* **43**: 3‐36 [PMID:18307107]

Kirk KL *et al*. (2011) A unified view of cystic fibrosis transmembrane conductance regulator (CFTR) gating: combining the allosterism of a ligand‐gated channel with the enzymatic activity of an ATP‐binding cassette (ABC) transporter. *J. Biol. Chem.* **286**: 12813‐9 [PMID:21296873]

Krämer BK *et al*. (2008) Mechanisms of Disease: the kidney‐specific chloride channels ClCKA and ClCKB, the Barttin subunit, and their clinical relevance. *Nat Clin Pract Nephrol* **4**: 38‐46 [PMID:18094726]

Kunzelmann K *et al*. (2011) Anoctamins. *Pflugers Arch.* **462**: 195‐208 [PMID:21607626]

Leblanc N *et al*. (2005) Regulation of calcium‐activated chloride channels in smooth muscle cells: a complex picture is emerging. *Can. J. Physiol. Pharmacol.* **83**: 541‐56 [PMID:16091780]

Muallem D *et al*. (2009) Review. ATP hydrolysis‐driven gating in cystic fibrosis transmembrane conductance regulator. *Philos. Trans. R. Soc. Lond., B, Biol. Sci.* **364**: 247‐55 [PMID:18957373]

Mulligan SJ *et al*. (2006) VRACs CARVe a path for novel mechanisms of communication in the CNS. *Sci. STKE* **2006**: pe42 [PMID:17047222]

Noy E *et al*. (2011) Combating cystic fibrosis: in search for CF transmembrane conductance regulator (CFTR) modulators. *ChemMedChem* **6**: 243‐51 [PMID:21275046]

Okada Y. (2006) Cell volume‐sensitive chloride channels: phenotypic properties and molecular identity. *Contrib Nephrol* **152**: 9‐24 [PMID:17065805]

Okada Y *et al*. (2009) Pathophysiology and puzzles of the volume‐sensitive outwardly rectifying anion channel. *J. Physiol. (Lond.)* **587**: 2141‐9 [PMID:19171657]

Patel AC *et al*. (2009) The role of CLCA proteins in inflammatory airway disease. *Annu. Rev. Physiol.* **71**: 425‐49 [PMID:18954282]

Planells‐Cases R *et al*. (2009) Chloride channelopathies. *Biochim. Biophys. Acta* **1792**: 173‐89 [PMID:19708126]

Plans V *et al*. (2009) Physiological roles of CLC Cl(‐)/H (+) exchangers in renal proximal tubules. *Pflugers Arch.* **458**: 23‐37 [PMID:18853181]

Puljak L *et al*. (2006) Emerging roles of chloride channels in human diseases. *Biochim. Biophys. Acta* **1762**: 404‐13 [PMID:16457993]

Pusch M *et al*. (2006) Channel or transporter? The CLC saga continues. *Exp. Physiol.* **91**: 149‐52 [PMID:16179405]

Riordan JR. (2005) Assembly of functional CFTR chloride channels. *Annu. Rev. Physiol.* **67**: 701‐18 [PMID:15709975]

Riquelme G. (2009) Placental chloride channels: a review. *Placenta* **30**: 659‐69 [PMID:19604577]

Sabirov RZ *et al*. (2009) The maxi‐anion channel: a classical channel playing novel roles through an unidentified molecular entity. *J Physiol Sci* **59**: 3‐21 [PMID:19340557]

Sloane PA *et al*. (2010) Cystic fibrosis transmembrane conductance regulator protein repair as a therapeutic strategy in cystic fibrosis. *Curr Opin Pulm Med* **16**: 591‐7 [PMID:20829696]

Verkman AS *et al*. (2009) Chloride channels as drug targets. *Nat Rev Drug Discov* **8**: 153‐71 [PMID:19153558]

## Connexins and Pannexins

### Overview

Gap junctions are essential for many physiological processes including cardiac and smooth muscle contraction, regulation of neuronal excitability and epithelial electrolyte transport [13, 17, 28]. Gap junction channels allow the passive diffusion of molecules of up to 1,000 Daltons which can include nutrients, metabolites and second messengers (such as IP_3_) as well as cations and anions. 21 connexin genes and 3 pannexin genes (which are structurally related to the invertebrate innexin genes) code for gap junction proteins in humans. Each connexin gap junction comprises 2 hemichannels or ‘connexons’ which are themselves formed from 6 connexin molecules. The various connexins have been observed to combine into both homomeric and heteromeric combinations, each of which may exhibit different functional properties. It is also suggested that individual hemichannels formed by a number of different connexins might be functional in at least some cells [39]. Connexins have a common topology, with four *α*‐helical transmembrane domains, two extracellular loops, a cytoplasmic loop, and N‐ and C‐termini located on the cytoplasmic membrane face. In mice, the most abundant connexins in electrical synapses in the brain seem to be Cx36, Cx45 and Cx57 [97]. Mutations in connexin genes are associated with the occurrence of a number of pathologies, such as peripheral neuropathies, cardiovascular diseases and hereditary deafness. The pannexin genes Px1 and Px2 are widely expressed in the mammalian brain [102]. Like the connexins, at least some of the pannexins can form hemichannels [13, 72].

**Table:** 

Nomenclature	Cx23	Cx25	Cx26	Cx30	Cx30.2
HGNC, UniProt	*GJE1*, A6NN92	*GJB7*, Q6PEY0	*GJB2*, P29033	*GJB6*, O95452	*GJC3*, Q8NFK1
Endogenous inhibitors	extracellular Ca^2+^ (blocked by raising external Ca^2+^)				
Inhibitors	carbenoxolone, flufenamic acid, octanol				

**Table:** 

Nomenclature	Cx30.3	Cx31	Cx31.1	Cx31.9	Cx32
HGNC, UniProt	*GJB4*, Q9NTQ9	*GJB3*, O75712	*GJB5*, O95377	*GJD3*, Q8N144	*GJB1*, P08034
Endogenous inhibitors	extracellular Ca^2+^ (blocked by raising external Ca^2+^)				
Inhibitors	carbenoxolone, flufenamic acid, octanol				

**Table:** 

Nomenclature	Cx36	Cx37	Cx40	Cx40.1	Cx43
HGNC, UniProt	*GJD2*, Q9UKL4	*GJA4*, P35212	*GJA5*, P36382	*GJD4*, Q96KN9	*GJA1*, P17302
Endogenous inhibitors	extracellular Ca^2+^ (blocked by raising external Ca^2+^)				
Inhibitors	carbenoxolone, flufenamic acid, octanol				

**Table:** 

Nomenclature	Cx45	Cx46	Cx47	Cx50	Cx59	Cx62
HGNC, UniProt	*GJC1*, P36383	*GJA3*, Q9Y6H8	*GJC2*, Q5T442	*GJA8*, P48165	*GJA9*, P57773	*GJA10*, Q969M2
Endogenous inhibitors	extracellular Ca^2+^ (blocked by raising external Ca^2+^)					
Inhibitors	carbenoxolone, flufenamic acid, octanol					

**Table:** 

Nomenclature	Px1	Px2	Px3
HGNC, UniProt	*PANX1*, Q96RD7	*PANX2*, Q96RD6	*PANX3*, Q96QZ0
Endogenous inhibitors	–	–	–
Inhibitors	carbenoxolone, flufenamic acid (little block by flufenamic acid)		
Comments	Px1 is unaffected by raising external Ca^2+^	Px2 is unaffected by raising external Ca^2+^	Px3 is unaffected by raising external Ca^2+^

### Comments

Connexins are most commonly named according to their molecular weights, so, for example, Cx23 is the connexin protein of 23 kDa. This can cause confusion when comparing between species ‐ for example, the mouse connexin Cx57 is orthologous to the human connexin Cx62. No natural toxin or specific inhibitor of junctional channels has been identified yet however two compounds often used experimentally to block connexins are carbenoxolone and flufenamic acid[85]. At least some pannexin hemichannels are more sensitive to carbenoxolone than connexins but much less sensitive to flufenamic acid [12]. It has been suggested that 2‐aminoethoxydiphenyl borate (2‐APB) may be a more effective blocker of some connexin channel subtypes (Cx26, Cx30, Cx36, Cx40, Cx45, Cx50) compared to others (Cx32, Cx43, Cx46, [8]).

### Further Reading

Cruciani V *et al*. (2006) The vertebrate connexin family. *Cell. Mol. Life Sci.* **63**: 1125‐40 [PMID:16568237]

Evans WH *et al*. (2006) The gap junction cellular internet: connexin hemichannels enter the signalling limelight. *Biochem. J.* **397**: 1‐14 [PMID:16761954]

Kandouz M *et al*. (2010) Gap junctions and connexins as therapeutic targets in cancer. *Expert Opin. Ther. Targets* **14**: 681‐92 [PMID:20446866]

MacVicar BA *et al*. (2010) Non‐junction functions of pannexin‐1 channels. *Trends Neurosci.* **33**: 93‐102 [PMID:20022389]

Mese G *et al*. (2007) Gap junctions: basic structure and function. *J. Invest. Dermatol.* **127**: 2516‐24 [PMID:17934503]

Shestopalov VI *et al*. (2008) Pannexins and gap junction protein diversity. *Cell. Mol. Life Sci.* **65**: 376‐94 [PMID:17982731]

Söhl G *et al*. (2005) Expression and functions of neuronal gap junctions. *Nat. Rev. Neurosci.* **6**: 191‐200 [PMID:15738956]

Zoidl G *et al*. (2010) Gap junctions in inherited human disease. *Pflugers Arch.* **460**: 451‐66 [PMID:20140684]

## Sodium leak channel, non‐selective

### Overview

The sodium leak channel, non selective (**NC‐IUPHAR tentatively recommends the nomenclature Na_Vi_2.1, W.A. Catterall, personal communication**) is structurally a member of the family of voltage‐gated sodium channel family (Na_v_1.1 ‐ Na_v_1.9) [47, 107]. In contrast to the latter, Na_Vi_2.1, is voltage‐insensitive (denoted in the subscript ‘vi’ in the tentative nomenclature) and possesses distinctive ion selectivity and pharmacological properties. Na_Vi_2.1, which is insensitive to tetrodotoxin (10 *μ*M), has been proposed to mediate the tetrodotoxin‐resistant and voltage‐insensitive Na^+^ leak current (I_L_‐Na) observed in many types of neurone [52]. However, whether Na_Vi_2.1 is constitutively active has been challenged [96]. Na_Vi_2.1 is widely distributed within the central nervous system and is also expressed in the heart and pancreas specifically, in rodents, within the islets of Langerhans [47, 52].

**Table:** 

Nomenclature	Na_vi_2.1
HGNC, UniProt	*NALCN*, Q8IZF0
Activators	Constitutively active (Lu *et al*., 2007), or activated downstream of Src family tyrosine kinases (SFKs) (Lu *et al*.,2009; Swayne *et al.*, 2009); positively modulated by decreased extracellular Ca^2+^ concentration (Lu *et al*., 2010) [52, 53, 54, 96]
Functional Characteristics	*γ* = 27 pS (by fluctuation analysis), *P*_Na_/*P*_Cs_ = 1.3, *P*_K_/*P*_Cs_ = 1.2, *P*_Ca_/*P*_Cs_ = 0.5, linear current voltage‐relationship, voltage‐independent and non‐inactivating
Channel blockers	Gd^3+^ (pIC_50_ 5.6), Cd^2+^ (pIC_50_ 3.8), Co^2+^ (pIC_50_ 3.6), verapamil (pIC_50_ 3.4)

### Comments

In native and recombinant expression systems Na_Vi_2.1 can be activated by stimulation of NK_1_(in hippocampal neurones), neurotensin (in ventral tegmental area neurones) and M3 muscarinic acetylcholine receptors (in MIN6 pancreatic *β*‐cells) and in a manner that is independent of signalling through G‐proteins [53, 96]. Pharmacological and molecular biological evidence indicates such modulation to occur though a pathway that involves the activation of Src family tyrosine kinases. It is suggested that Na_Vi_2.1 exists as a macromolecular complex with M3 receptors [96] and peptide receptors [53], in the latter instance in association with the protein UNC‐80, which recruits Src to the channel complex [53, 103]. By contrast, stimulation of Na_vi_2.1 by decreased extracellular Ca^2+^ concentration is G‐protein dependent and involves a Ca^2+^‐sensing G protein‐coupled receptor and UNC80 which links Na_vi_2.1 to the protein UNC79 in the same complex [54]. Na_Vi_2.1 null mutant mice have severe disturbances in respiratory rhythm and die within 24 hours of birth [52]. Na_vi_2.1 heterozygous knockout mice display increased serum sodium concentrations in comparison to wildtype littermates and a role for the channel in osmoregulation has been postulated [92].
